# Ionic Transport in CsNO_2_-Based Nanocomposites with Inclusions of Surface Functionalized Nanodiamonds

**DOI:** 10.3390/nano11020414

**Published:** 2021-02-05

**Authors:** Yulia G. Mateyshina, Dmitriy V. Alekseev, Nikolai F. Uvarov

**Affiliations:** 1 Institute of Solid State Chemistry and Mechanochemistry SB RAS, Kutateladze 18, 630090 Novosibirsk, Russia; d.alekseev1@list.ru (D.V.A.); uvarov@solid.nsc.ru (N.F.U.); 2 Department of Natural Sciences, Novosibirsk State University, Pirogova, 2, 630090 Novosibirsk, Russia; 3 Faculty of Mechanical Engineering and Technologies, Novosibirsk State Technical University, Prospect Karla Marksa 20, 630073 Novosibirsk, Russia

**Keywords:** ionic conductivity, electrochemical cell, solid composite electrolytes, nanodiamonds

## Abstract

Composite solid electrolytes (1 − x)CsNO_2_-xND, where ND are nanodiamonds, including those after liquid-phase and gas-phase oxidation and reduction functionalization, were prepared, and their properties investigated by XRD, analysis of BET nitrogen adsorption isotherms, IR spectroscopy and impedance spectroscopy. The electrical conductivity of composites (1 − x)CsNO_2_-xND obeys the Arrhenius dependence and has a maximum at x = 0.95 regardless of the ND pretreatment. It was found that the conductivity depends on the mode of functionalization of the ND surface, as well as on the processing time. The electrical conductivity of composites with ND, processed by the gas-phase method, is 1.5–2.6 times higher than that of composites with initial ND, in which the conductivity is 2 orders of magnitude higher than that of pure cesium nitrate. Thus, the possibility of using ND as an effective heterogeneous additive for the preparation of composite solid electrolytes, including cesium nitrite, has been demonstrated for the first time.

## 1. Introduction

A diamond is an allotropic modification of carbon, known to humankind since ancient times. It consists of sp^3^-hybridized carbon atoms and crystallizes in the corresponding cubic system, the densest atomic system among carbon polymorphs. It has a wide bandgap and high radiation resistance. Diamonds are chemically inert and stable at temperatures below 850 °C. Due to these properties, diamonds find application in various fields of science and technology. Diamonds with characteristic sizes from several to hundreds of nanometers are called nanodiamonds or ultradispersed diamonds (NDs). At present, NDs are commercially manufactured by a detonation synthesis technique. The main application fields of detonation NDs are medicine and pharmaceuticals, catalysts support, functional coatings, additives to lubricants and composites. A remarkable feature of NDs is the very small crystal size of 4–6 nm [1]. ND particles may be obtained under nonequilibrium conditions at high pressures (~20 GPa) and temperatures (~3000 K) in a stable diamond phase. For most applications of ND, the outer surface functional layer plays an important role. In the course of synthesis, ND particles are subjected to a chemical purification procedure resulting in coating the surface with oxygen-containing and other functional groups. In the case of detonation synthesis, the purification of the nanoscale diamond is most often performed using oxidation in a mixture of mineral acids and/or oxidation in the air [2,3,4,5,6]. Both treatments lead to the formation of carbonyl and carboxyl groups on the surface of the diamond. Due to the use of water or ice for cooling during the detonation process, hydroxyl groups also form on the ND surface [7,8,9,10,11,12]. Carboxyl groups are prevailing functional groups on the ND surface. The carboxyl group is the most oxidized state of carbon on the surface, where the carbon atom is attached by only one bond to the diamond lattice. With the maximum oxidation of the nanodiamond surface, the surface is filled mainly by carboxyl groups. This is inevitably accompanied by a loss of material and a decrease in the size of the diamond core [13]. Complete oxidation of the ND surface can be also carried out both in a mixture of concentrated mineral acid oxidizing agents and in the presence of other oxidizing agents [4,11,14,15]. Another method for the introduction of oxygen-containing groups onto the ND surface is oxidation in the air. Gogotsi and colleagues [16] reported a method for the functionalization of carbon by oxidation in air at elevated temperatures above 450 °C. This method allows one to control the ratio of carbon atoms in sp^3^ and sp^2^ hybridization states. At such temperatures (> 450 °C), all types of nanoscale carbon undergo oxidation; therefore, the ND particle also begins to oxidize. In a favorable temperature range of nearly 425 °C, sp^2^, carbon atoms are selectively oxidized, and a large number of oxygen-containing groups are formed on the ND surface. After air treatment, most of the surface groups are carboxyl ones—COOH— however, in addition to them, there are ketone, carbonyl, ether and hydroxyl groups. It is worth noting that air oxidation may be used to deliberately reduce the size of nanoscale diamond particles.

As functional groups on the ND surface, not only carboxyl and carbonyl groups, but also hydroxyl ones, are important, since they open up a wide range of subsequent reactions. Hence, it is obvious that the coating of a nanodiamond surface with a large number of OH groups is one of the most studied methods of surface homogenization [17,18,19,20]. It should be noted that all the methods indicated in the literature do not lead to the complete removal of -C=O groups from the ND surface. Most reactions using reducing agents lead to the formation of hydroxyl groups. Early studies showed that decarbonylation and decarboxylation take place under the treatment of NDs with hydrogen at high temperatures or in plasma [19]. Thus, the formation of C-H bonds on a diamond surface is possible only as a result of a direct reaction with elemental hydrogen. For example, such a reaction proceeds when the hydrogen flow passes through the ND powder layer at elevated temperatures. This method was proposed by Spitsyn et al. [13]. However, the authors noted that in addition to the formation of C-H bonds, an increase in the number of OH groups was observed at temperatures of nearly 800 °C. In organic chemistry, the hydrogenation of unsaturated hydrocarbons is usually carried out using a metal catalyst (palladium, platinum, nickel). Nevertheless, the catalytic hydrogenation of NDs is rarely mentioned, most likely due to the difficulty in the separation of catalysts from nanoparticles. Usually, mineral acid-oxidizing agents are used to remove catalysts, which in turn, lead to reoxidation of the diamond surface. For diamond films, the treatment in hydrogen plasma is a generally accepted method [21,22,23]. Thus, to date, there are several methods for ND surface treatment that make it possible to modify surface layer properties, for example, to provide hydrophobicity or hydrophilicity of the ND surface. It is also worth noting that reductive surface functionalization provides surface accessibility that facilitates the conversion of carboxyl and carbonyl surface groups into alkyl groups.

Earlier we carried out a comparative study of ionic conductivity in a series of alkali metal nitrites [24,25]. The experimental results were interpreted in terms of the classical model of the formation of Schottky defects in ionic crystals, when cation vacancies are the dominant charge carriers. It was shown that in a series of alkaline nitrites, both defect formation and enthalpies of migration decrease with increasing the cation radius, which leads to increased conductivity. The most unusual results were obtained for cesium nitrite, which showed a fairly high conductivity in the range from 5 × 10^−8^ S/cm at 25 °C to 10^−3^ S/cm at 350 °C. A further increase in the conductivity values in cesium nitrite was observed in composite solid electrolytes obtained by the introduction of heterogeneous additive into the CsNO_2_ matrix. Nanocrystalline alumina [26], magnesium oxide [26], SiO_2_ [26], SnO_2_ [27] and MgAl_2_O_4_ [28] were used as the heterogeneous additives. Recently, it was found that NDs may be used as a heterogeneous dopant for the preparation of nanocomposite solid electrolytes AgI-ND [29]. To date, besides the paper mentioned, no information is known on the use of such non-oxide additives as NDs for the preparation of composite solid electrolytes. Furthermore, it has not been investigated how surface modification of ND influenced transport properties of the composites. The aim of this work is to study the effect of oxidative and reductive functionalization of ND surface on the transport properties of composite solid electrolytes CsNO_2_-ND.

## 2. Materials and Methods

Powder of ND (UDA-C type with the specific surface area Ss = 320 ± 20 m^2^/g) was produced by detonation technique in the Federal Research and Production Center “Altai”, Biysk, Russia. Functionalization of the ND surface was done as follows:

Liquid-phase oxidative functionalization of ND powder was carried out in a mixture of concentrated nitric and sulfuric acids (volume ratio = 1: 4) at a temperature of 120 °C under constant stirring. At certain time intervals (10, 20, and 30 min, 1, 2, 3 and 48 h), portions of the sample were extracted, transferred to a glass and treated by a 0.1 M NaOH solution by slow dropping to a slightly acidic medium (pH = 5–6). After that, the samples were washed with distilled water until a neutral medium (pH = 6–7) was obtained, and the precipitates were separated by centrifugation.

The gas-phase oxidative functionalization of the ND surface was carried out in the air, where oxygen was the oxidizing agent, at T = 200 °C for 19 and 27 h, and at T = 300 °C for 10 and 15 h. The oxidation process was carried out in a muffle furnace.

Gas-phase reductive functionalization of NDs was carried out in a tubular furnace in a stream of hydrogen at a temperature of 650 °C with a heating rate of 10 deg/min. For sampling, the furnace was cooled and part of the product was removed (0.5, 2, and 4 h).

The crystal structure of the obtained composites was analyzed using the X-ray diffraction (XRD) technique. XRD patterns were recorded on a D8 Advance X-ray diffractometer (Bruker, Ettlingen, Germany) with θ-θ geometry equipped with a one-dimensional Lynx-Eye detector and Kα-filter using Cu-Kα radiation in the angle interval of 10° < 2θ < 90° with a step size of ∆2θ = 0.0195° and a counting time of 35.4 s per step.

The specific surface area of ND samples was determined from the analysis of nitrogen adsorption BET isotherm obtained on a Termosorb TPD 1200 analyzer (Catakon, Novosibirsk, Russia). The samples were heated at 200 °C for 60 min before adsorption measurements.

Results obtained for ND are well reproduced. Experiments on acidic surface oxidation of nanodiamonds were carried out three times to reproduce the results and obtain functionalized nanodiamond. Both gas-phase modifications were carried out 2 times. All the samples were characterized by methods of XRD and BET adsorption isotherms.

IR spectroscopy was carried out using an EXCALIBUR 3100 IR Fourier spectrometer (Varian, Walnut Creek, CA, USA) in the range ω = 500–4000 cm^−1^. The absorption bands were assigned according to [30]. SEM analysis of the samples was carried out using a scanning electron microscope HITACHI TM1000 (Tokyo, Japan).

For the synthesis of composite solid electrolytes (1 − x)CsNO_2_-xND, CsNO_2_ (99.9% pure, produced at Rare Metals Plant, Ltd., Novosibirsk, Russia) and initial and functionalized NDs were used. Composites (1 − x)CsNO_2_-xND (x is the molar ratio, 0 < x < 1) were prepared from preliminary dried components. The initial powders were mixed, heated at 200 °C for 180 min and pressed at 400 MPa together with two silver electrodes into pellets with a thickness of 0.1–0.4 cm and a diameter of 0.5–0.6 cm. Relative densities of the pellets were more than 90% of the theoretical ones.

Conductivity was measured in a two-electrode cell in the vacuum in the temperature range 25–160 °C in the stepwise isotherm mode using a Hewlett Packard HP 4284A precision LCR meter (Tokyo, Japan) in the frequency range of 20 Hz^−1^ MHz at the AC voltage of 10 mV. The values of electrical conductivity were calculated from the analysis of the impedance plots Z′ = f (Z”). The heating rate was 30 deg/h, the exposure time at each temperature before the measurement was 10 min. The data were well reproduced when measured in 4 subsequent heating-cooling cycles. Conductivity measurement was carried out in parallel on 2–3 pellets with the same composition but different in size.

## 3. Results and Discussion

### 3.1. Initial Nanodiamonds

**Figure 1:**
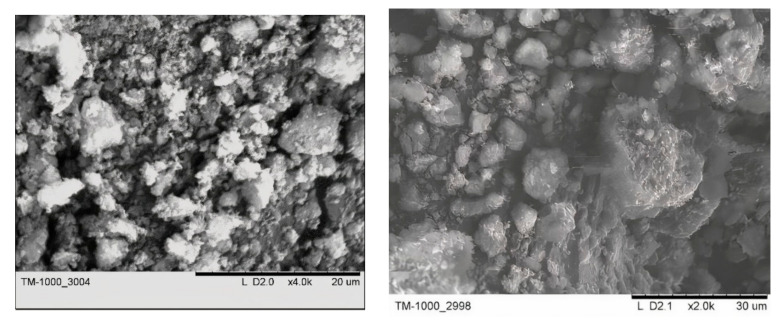
Typical SEM images of nanodiamonds.

The initial ND powder is weakly crystallized particles ranging in size from several nanometers to aggregated particles of several tens of microns in size (Figure 1).

The specific surface area of initial NDs was determined from the BET adsorption isotherms of nitrogen as S_s_ = 293 m^2^/g. The average particle size, R_BET_ = 7.73 nm, was estimated from the BET data assuming that crystallites have a cubic shape. Analysis of XRD data showed that only broadened reflections attributed to the diamond phase (symmetry space group Fd3m, the lattice parameter a_c_ =3.5597 Å) present in the diffraction pattern. The size of the coherent scattering regions (CSR), which may be related to single ND crystals, R_XRD_ = 7.49 nm, are in good agreement with the R_BET_ values.

### 3.2. The Effect of ND Surface Treatment on the Crystal Structure and Specific Surface Area

**Figure 2:**
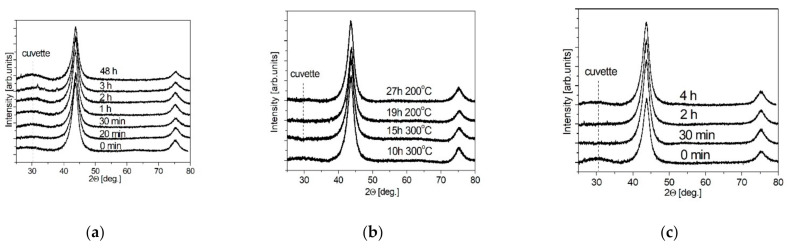
XRD patterns of the samples ND^acid^ (**a**), ND^air^ (**b**) and ND^H2^ (**c**).

The effect of ND surface functionalization was studied by the XRD method. The results are shown in Figure 2 for samples subjected to liquid-phase oxidation ND^acid^ (Figure 2a), gas-phase oxidation ND^air^ (Figure 2b) and gas-phase reduction ND^H2^ (Figure 2c). X-ray diffraction patterns contain two reflections ((111), (220)), which are related to the diamond structure. No reflections attributed to impurity phases were found.

Experimental values of CSR estimated using the Scherrer formula and specific surface area are listed in Table 1. CSR values, R_XRD_, in functionalized NDs were 4.2–4.6 nm for liquid-phase oxidation, 4.0–4.7 nm for gas-phase oxidation and 3.8–5.1 nm for gas-phase reduction. For all types of ND functionalization, CSR values increased with the functionalization time. During oxidation, the bulk diamond phase “burns out”, since the treatment was carried out either in a strongly oxidizing environment or at high temperatures (for example, in the method of reduction with hydrogen). The initial ND powder is characterized by a high specific surface area S_s_ = 293 m^2^/g and an average pore size R_pore_ = 2.9 nm. The liquid-phase oxidative functionalization of nanodiamonds leads to a gradual decrease in the specific surface area to 110 m^2^/g after the treatment over 48 h, and the pore size changes to R_pore_ = 2.4 nm.

On the contrary, oxidation in a gas-phase medium leads to an increase in the specific surface area of ND up to 352 m^2^/g, indicating a noticeable burnout of carbon [23]. A further increase in the time and/or temperature of the oxidative gas-phase functionalization of the surface of nanodiamonds leads to a decrease in S_s_ and is not considered in this work. Reductive functionalization for 4 h slightly increases the specific surface area up to the value of S_s_ = 317 m^2^/g

#### Investigation of the Surface of Nanodiamonds by IR Spectroscopy

**Figure 3:**
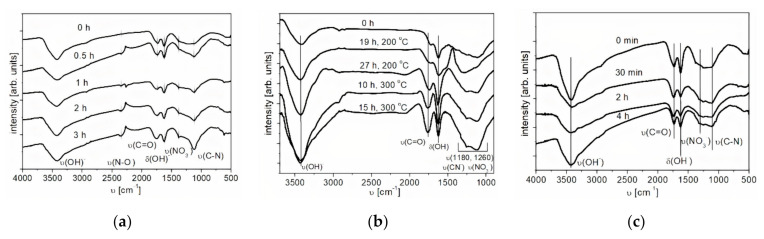
IR spectrum of nanodiamonds (NDs) oxidized by liquid-phase (**a**) and gas-phase (**b**) methods and reduced by H_2_ (**c**); υ—stretching vibrations, δ—deformation vibrations.

The investigation of the surface functional groups in initial and functionalized NDs was carried out by IR spectroscopy. The IR spectra of the initial ND and the treated samples are shown in Figure 3a–c. A characteristic feature of the infrared absorption spectra of the samples is the presence of a wide absorption band in the range from 3600 to 3000 cm^−1^ with a maximum at 3464 cm^−1^, caused by the stretching vibrations of adsorbed water molecules and surface OH-groups. The absorption band at 1568 cm^−1^ can be attributed to bending vibrations of O-H groups. Moreover, in the IR spectrum of the sample treated in acids, stretching vibrations of N-O (1300–1200 cm^−1^) were identified, the intensity of which increases with an increase in the time of acid treatment of ND. In addition to the described absorption bands, in the spectra of oxidized nanodiamonds, one can observe the presence of an absorption band at 1764 cm^−1^, which is related to stretching vibrations of carbonyl groups. Increasing the time of oxidative functionalization in air leads to an increase in the intensity of peaks related to carbonyl groups, which confirms the oxidation of nanodiamonds. The increase in the intensity of the peak attributed to stretching vibrations of the (C-N) bond (υ = 1160 cm^−1^) also confirms this fact.

The spectrum of the reduced nanodiamonds contains absorption bands in the range from 3600 to 3000 cm^−1^, with a maximum at 3464 cm^−1^, which are responsible for the stretching vibrations of hydroxyl groups, and at 1568 cm^−1^ for the bending vibrations of O-H groups (Figure 3c). Furthermore, in the IR spectrum of the sample, stretching vibrations of N-O (1300–1200 cm^−1^) were identified, the intensity of which decreases with an increase in the time of the reduction treatment of NDs. The presence of absorption bands at 1764 cm^−1^ indicates the presence of stretching vibrations of carbonyl groups. On the whole, for the samples of reduced NDs, the same absorption bands were observed as for the initial NDs. With an increase in the functionalization time, the intensity of the peaks related to carbonyl groups (1750 cm^−1^) decreases.

### 3.3. Transport Properties of Composites (1 − x)CsNO_2_-xND

The initial cesium nitrite has a high ionic conductivity of 1.3 × 10^−4^ S/cm at 265 °C (E_a_ = 0.82 eV) [24]. A detailed study of its transport properties was carried out in [25], and it was shown that the conductivity of pure nitrite is ionic and governed by cations.

**Figure 4:**
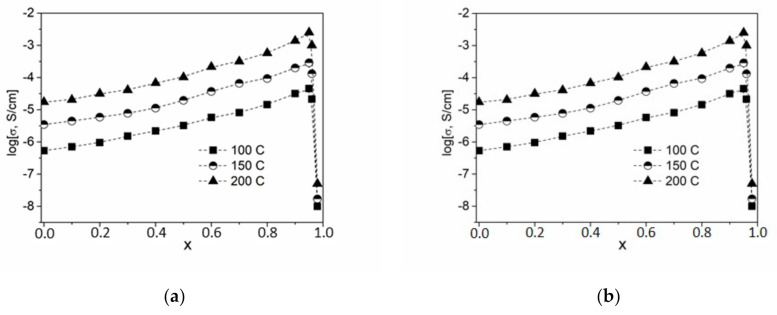
Temperature (**a**) and concentration (**b**) dependences of conductivity for composites (1 − x)CsNO_2_–xND. Points 1, 2, 3, 4, 5 and 6 correspond to the ND concentration of x = 0, 0.3, 0.7, 0.8, 0.9 and 0.95, respectively.

In this work, composites were synthesized in a wide range of concentrations using the ceramic technique, and their transport properties were studied by impedance spectroscopy. For each temperature, impedance hodographs were plotted in Nyquist coordinates. The conductivity values σ are well reproduced in heating–cooling cycles and are stable during long-term exposure in a vacuum under isothermal conditions. The temperature dependence of the electrical conductivity of the studied samples (1 − x)CsNO_2_—xND is plotted in Figure 4a. Experimental conductivity data are fairly well described by Arrhenius dependences σT = A exp (−E_a_/kT) over the entire temperature range under study.

Concentration dependences log (σ) = f(x) are shown in Figure 4b. It can be seen that the dependence passes through a maximum at x = 0.95 and strongly decreases at higher concentrations of NDs. Thus, the conductivity of CsNO_2_-ND composites increased by nearly 2 orders of magnitude (at x = 0.95, σ = 4.3 × 10^−3^ at 200 ℃) compared to pure ionic salt (at x = 0, σ = 1.8 × 10^−5^ at 200 ℃). The value x = 0.95 corresponds to a volume fraction of NDs in the composites equal to nearly f = 55%. Such conductivity behavior is typical for composite solid electrolytes, the conductivity of which is caused by ionic transport via interfaces between the ionic salt and the heterogeneous additive.

The physical reason for the conductivity increase is the formation of the interface regions enriched in the defects [31]. In turn, the formation of such regions is limited by the wettability of the ND surface with ionic salt CsNO_2_. Therefore, a further increase in the conductivity can be expected due to an improvement in the surface wettability of the NDs. To ensure good wettability between the components of the system, one can modify the ND surface with active functional groups. Since the ND surface may contain a wide spectrum of various groups, surface modification may be regarded as an efficient root for the control of wettability and, thereby, ion transport properties of ND-based composites. Such experiments were done, and the results are presented below.

#### Transport Properties of Composites (1 − x)CsNO_2_-xND with Functionalized ND

Solid composite electrolytes were synthesized with functionalized ND and their transport properties were investigated. Transport properties of composites with a maximum conductivity of 0.1CsNO_2_-0.9ND were compared. The results of the comparison are presented in Table 2.

The comparison shows that, in general, the functionalization of the ND surface affects the conductivity values (Table 2). In all cases, after functionalization, conductivity of composites 0.1CsNO_2_-0.9ND increased by 1.5–2.6 times. The highest conductivity value, 5.0 × 10^−3^ at 200 °C was observed for the composite containing oxidized sample ND^air^ treated using the method of gas-phase oxidation in the air for 15 h. Since ionic conductivity in composites occurs along with CsNO_2_/ND interfaces, this effect can be caused by a change in the specific surface area of NDs as a result of the functionalization. The highest values of specific surface area were obtained for ND^air^ and ND^H2^ samples obtained by gas-phase treatment by oxidizing in the air (S_s_ = 352 m^2^/g) and in the hydrogen atmosphere (S_s_ = 317 m^2^/g). The samples with the highest S_s_ values have the highest conductivities.

**Figure 5:**
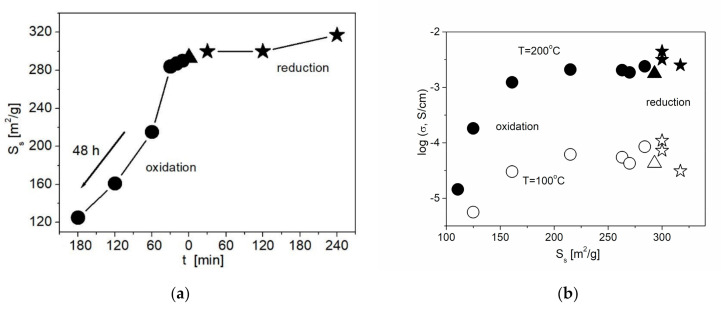
Change in the specific surface area of NDs (**a**) and the conductivity of 0.1CsNO_2–_ 0.9ND composites (**b**) with the treatment time for various methods of ND functionalization. Data for samples containing unmodified NDs, ND^Acid^ and ND^H2^ are denoted by triangles, circles and stars, respectively.

On the other hand, liquid-phase oxidation of the ND surface leads to a decrease in S_s_ (Figure 5a). The decrease in the specific surface area is likely to be caused by the increase in the concentration of functional groups forming a thick core on the surface of ND. Nevertheless, even with a decrease in S_s_, a slight increase in the conductivity values is observed compared to the composite with the initial ND at a not long time of the acid treatment. The surface of the initial nanodiamonds is generally weakly polar, and the adhesion of the ionic salt to such a surface is low. An increase in the surface polarity of nanodiamonds due to the formation of functional groups (carboxyl, carbonyl, etc.) leads to an increase in salt adhesion, which in turn leads to an increase in conductivity by 1.5 times. The increase in the treatment time leads to a decrease in both S_s_ and conductivity values. A general correlation between the conductivity and the functionalization time is shown in Figure 5b. Due to the effect of two controversial factors, the increase in the wettability and the decrease in the specific surface area, conductivity of the samples containing ND^Acid^ additive tends to go through a smooth maximum.

## 4. Conclusions

In this work, the ionic conductivity of CsNO_2_-ND with detonation nanodiamonds as an additive was studied. The ND additives were treated in oxidative and reducing media for modification of their surface. The effect of ND surface treatment on properties of ND was investigated by methods of XRD, analysis of BET nitrogen adsorption isotherms and IR spectroscopy. It was found that oxidative treatment in acidic water solutions leads to the decrease in the specific surface area, where the gas phase treatment in oxidative and reducing atmosphere results in the increase in S_s_ values. The conductivity of (1 − x)CsNO_2_—xND composites obeys Arrhenius dependence and goes through a maximum at x = 0.95 corresponding to the volume fraction of f = 55%, irrespectively of the preliminary treatment of NDs. It was found that the conductivity depends on the method of the ND surface functionalization, the modifying agent and the treatment time. In general, the maximum conductivity values correlate with the specific surface area of ND. The conductivity of the composites containing ND treated by gas-phase methods are 1.5–2.6 times higher than those containing initial ND. The dependence of the conductivity on the specific surface has a non-linear character. In particular, liquid-phase oxidation of the ND surface leads to a decrease in S_s_, which is likely to be caused by the increase in the concentration of functional groups forming a thick core on the surface of ND. Nevertheless, even with a decrease in S_s_, a slight increase in the conductivity values is observed compared to the composite with the initial ND at a not long time of acid treatment. The acid treatment leads to the formation of functional groups and to the increase in salt adhesion, which in turn results in an increase in conductivity by 1.5 times. The increase in the treatment time leads to a decrease of both S_s_ and conductivity values. Due to the effect of two controversial factors, the increase in the wettability and the decrease in the specific surface area, the conductivity of the samples containing ND^Acid^ additive tends to go through a smooth maximum. Thus, it was first demonstrated that ND may be used as an effective heterogeneous additive for the preparation of composite solid electrolytes, including cesium nitrite. Moreover, the conductivity of the composites may be further increased by ND surface functionalization.

## Figures and Tables

**Table 1:** The value of the dimensions of the CSR and the specific surface area.

Functionalization Condition, h			CSR by Scherrer (± 1.0), nm	S_s_, m^2^/g
Initial ND		0	7.5	293 ± 20
Liquid-phase oxidation (ND^acid^)		0.17	4.6	290 ± 20
		0.33	4.4	287 ± 20
		0.5	4.4	284 ± 20
		1	4.3	215 ± 20
		2	4.3	161 ± 20
		3	4.2	125 ± 10
		48	4.2	110 ± 10
Gas-phase oxidation (ND^air^)	T = 200 °C	19	4.2	333 ± 20
		27	4.2	308 ± 20
	T = 300 °C	10	4.7	322 ± 20
		15	4.0	352 ± 20
Gas-phase reduction (ND^H2^)		0.5	5.1	300 ± 20
		2	4.3	294 ± 20
		4	3.8	317 ± 20

**Table 2:** Comparison of the conductivity values for pure CsNO_2_ and composite solid electrolytes 0.1CsNO_2_-0.9ND with initial ND and ND functionalized by different methods.

	CsNO_2_	0.1CsNO_2_-0.9ND Composites			
		Initial ND	ND^Acid^	ND^air^	ND^H2^
**T, °C**	**σ, S/cm**				
**150**	3.2 × 10^−6^	3.8 × 10^−4^	5.6 × 10^−4^	1.0 × 10^−3^	8.3 × 10^−4^
**200**	1.1 × 10^−5^	1.9 × 10^−3^	3.2 × 10^−3^	5.0 × 10^−3^	4.8 × 10^−3^
